# Changes in Cytokinin Concentration in Gall Tissues Induced by *Dryocosmus kuriphilus* and the Spatiotemporal Distribution Characteristics of Cytokinin in the Body of *Dryocosmus kuriphilus*

**DOI:** 10.3390/insects17060572

**Published:** 2026-05-30

**Authors:** Ankang Lv, Xiaohui Yang, Yang Zeng, Daohong Zhu

**Affiliations:** 1 Laboratory of Insect Behavior and Evolutionary Ecology, College of Life Science and Technology, Central South University of Forestry and Technology, Changsha 410004, China; yl240019@126.com (A.L.); zengyangsile@163.com (Y.Z.); 2 College of Life Sciences, Hunan Normal University, Changsha 410004, China; xhyang@hunnu.edu.cn

**Keywords:** *Dryocosmus kuriphilus*, cytokinins, coevolution

## Abstract

This study aimed to characterize the sources and spatio-temporal distribution patterns of cytokinins in *Dryocosmus kuriphilus* and its induced galls, providing observational data on possible gall-inducing mechanisms. Specifically, the contents of six cytokinins (TZR, DZR, *cis*-Zeatin, *trans*-Zeatin, IP, and IPR) in the epidermal layer (IPR: 3.92 ± 0.15 ng/g), outer protective layer, inner protective layer, and nutritive layer of galls, as well as in *D. kuriphilus* at different developmental stages, were quantified by UPLC-MS/MS. The results showed that IPR (63.12 ± 2.55 ng/g) was relatively high in larvae. This observational finding is consistent with the hypothesis that cytokinins in *D*. *kuriphilus* may originate from the insect itself or from plant sources. Elevated cytokinin levels were found in plant galls compared to normal tissues.

## Simple Summary

This study analyzed the spatio-temporal distribution patterns of six cytokinins in the chestnut gall wasp and its induced galls. The results revealed that cytokinin concentrations were relatively high in the larvae, while normal plant tissues lacked detectable zeatin-type cytokinins. Additionally, cytokinin levels in the galls were significantly higher than those in normal plant tissues. These findings provide observational data for understanding the cytokinin-mediated manipulation of host plant development by the chestnut gall wasp and lay the groundwork for future studies on gall formation and potential hormone-based pest management strategies.

## 1. Introduction

*Dryocosmus kuriphilus* (Hymenoptera: Cynipidae), members of the tribe Cynipini, are significant pests that threaten almost all chestnut trees (*Castanea* spp.), with *C. pumila* being the only known exception [1]. The pest prevent or inhibit the development of normal shoots and often cause abnormal growths, known as galls, in plants [2]. In the long term, the gall wasp can cause an 80% yield loss in heavily infested trees while compromising chestnut quality [3,4]. Parthenogenetic females oviposit into plant tissues shortly after summer emergence from galls, and the newly hatched larvae overwinter within these tissues [5]. Eggs and the first-instar larvae overwinter within the buds. After overwintering, the larvae commence feeding, causing damage and ultimately inducing gall formation. The rapidly dividing and atypically differentiating cells in galls point to potential hormonal control of their developmental programs. Research suggests that plant hormones, particularly cytokinins, play a significant role in insect-induced gall formation [6,7,8]. Understanding the spatiotemporal pattern of cytokinins in galls may help elucidate how gall wasps manipulate host plant development.

Microscopic examination of gall tissues reveals that gall formation involves initial extensive cell division and that cellular enlargement takes place progressively in the growth, differentiation, and maturity stages [9,10]. Previous results suggest that gall tissue is free from feedback regulation and more sensitively transduces the cytokinin signals [11]. Certain insects can manipulate host plant physiology through cytokinin transfer, altering source/sink relationships to enhance nutritional quality and mitigate fitness costs [12,13]. Gall development ceases if the larva inside the gall dies. Therefore, it is reasonable to conclude that both the initiation and continuous growth of the gall depend on secretions produced by the gall-forming insect. The rapid growth and specific differentiation of gall cells suggest the existence of hormonal control over their activities [14,15].

Cytokinins (CKs), adenine derivatives with isoprenoid or aromatic side chains, are phytohormones that have been strongly suggested to be involved in gall induction by insects and are commonly identified in insect secretions or glands associated with gall formation [16]. However, direct evidence of their causal role remains to be demonstrated. Importantly, CKs are not restricted to gall-inducing insects; they are also abundant in non-gall-inducing insects, where they may play roles in modulating nutrient flux and plant defenses during herbivory [3,14]. A systematic survey by Tokuda et al. detected CKs in diverse arthropod species, including both gall-inducing and non-gall-inducing insects [17]. Among gall-inducing species, the Chinese sumac aphid *Schlechtendalia chinensis* (Hemiptera) on *Rhus chinensis*, *Tamalia coweni* (Hemiptera) on *Arctostaphylos* spp., and the goldenrod ball-gall fly *Eurosta solidaginis* (Tephritidae) on *Solidago* spp. all contain detectable CKs, despite not all being gall-inducing in the same manner [14,18,19]. The larval stages of gall-inducing insects exhibit significantly higher concentrations of specific cytokinins—including isopentenyladenine (IP), isopentenyladenosine (IPR), *trans*-Zeatin (TZ), and *cis*-Zeatin (CZ)—than the surrounding gall tissue and host plant leaves, in which their levels are substantially lower [20,21].

In plants, two cytokinin biosynthetic pathways are known: the tRNA degradation pathway (producing mainly CZ-type) and the de novo pathway catalyzed by ATP/ADP-dependent isopentenyltransferase (IPT). In the de novo pathway, iP ribotides are hydroxylated by *CYP735A* to tZ-type nucleotides, and LOG proteins catalyze the final conversion of nucleotides to active free bases. Insects differ markedly from plants. Evidence for insect cytokinin biosynthesis pathways remains limited. A non-peer-reviewed study on *Drosophila melanogaster* reported that the fly genome lacks adenylate-IPT homologs and suggested that the tRNA degradation pathway may be a major route for cytokinin production [22]. However, whether this finding can be generalized to other insects, including gall wasps, awaits further investigation. Unlike plants where tRNA degradation yields mainly CZ-type cytokinins, in animals (including insects) the same pathway produces predominantly isopentenyladenosine (IPR) and its derivatives [14,17]. Functional genetic studies in *Drosophila* have confirmed that *tRNA-IPT* genes contribute significantly to the total cytokinin pool [22]. For side-chain hydroxylation, insects possess a distinct enzyme, *CYP6CY6v2*, which accepts a broader range of substrates than plant *CYP735A* [23]. By contrast, the activation step via *LOG* homologs has not been functionally characterized in insects, and the full biosynthetic network remains partially unknown. A unique bifunctional enzyme, *CpIPT-LOG*, found in *Claviceps purpurea*, is involved in the de novo synthesis of CKs and their interconversion between nucleotide and free-base forms. Its *LOG* domain directly converts inactive cytokinin nucleotides (e.g., IPRMP and TZMP) into their bioactive free-base forms (IP and TZ) [24].

Researchers observe that injecting single or mixed plant hormones induces partial gall-like phenotypes, suggesting that gall formation may be mediated by exogenous CKs [7,15,25]. As research progresses, a growing body of evidence substantiates CKs’ pivotal involvement in gall formation. The presence of CKs in *D*. *kuriphilus* larvae was detected quite early, implying a possible relationship with gall formation, although precise quantitative analysis was lacking [26,27,28]. In fact, galls, as a special type of plant “organs”, exhibit spatial and temporal heterogeneity. While research has examined CKs in *D*. *kuriphilus*, quantitative variations in CKs in galls versus larvae, or among different tissues and developmental stages have not been reported. This study focuses on the chestnut gall wasp as an important forest pest, investigating CK distribution patterns across insect-gall interactions, with three objectives: (1) What are the contents and profiles of various CKs (including CZ, TZR, IP, IPR, DZR, TZ) in different developmental stages of *D. kuriphilus* (larvae, pupae, adults) and in distinct tissue layers of the induced galls (epidermis, outer protective layer, inner protective layer, nutritive layer)? (2) How do CK levels in gall tissues compare with those in normal plant tissues? (3) Based on the observed distribution patterns, can we infer whether the CKs originate from insect synthesis, plant accumulation, or both?

## 2. Materials and Methods

### 2.1. Plant and Insect Materials

The chestnut gall wasp typically induces galls as larvae, and the larvae are extremely small in fresh weight (<0.005 g). Therefore, a large number of individuals need to be collected to obtain sufficient tissue for CK analysis. Gall specimens and associated *Dryocosmus kuriphilus* (Hymenoptera: Cynipidae) were collected from Changsha County, Changsha City, Hunan Province, China. Samples were immediately frozen in liquid nitrogen, stored at −80 °C, and transported on dry ice for analysis.

**Figure 1:**
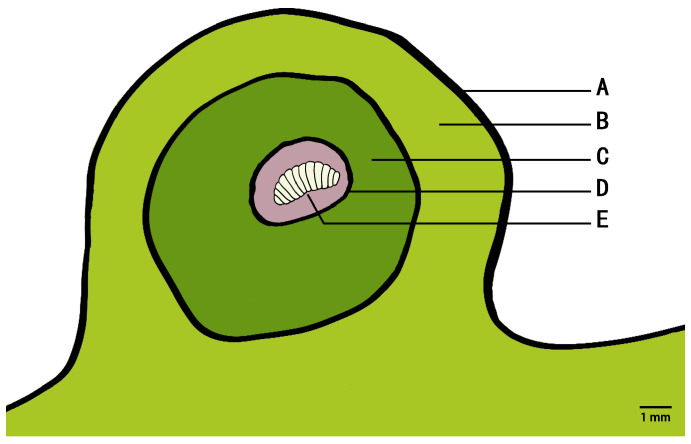
Cross-sectional layer diagram of *Dryocosmus kuriphilus* galls. A: epidermis; B: outer protective layer; C: inner protective layer; D: nutritive layer; E: larvae.

Our previous research has revealed that within the protective layer of chestnut gall wasp galls, two distinct tissues can be identified: the parenchyma layer and the sclerenchyma layer. However, it remains unclear whether the CK concentration differs between these two layers. Therefore, in this experiment, the gall was divided into the epidermis, the inner protective layer (parenchyma), the outer protective layer (sclerenchyma), and the nutritive layer (Figure 1). Normal tissue: Healthy leaves collected from normally growing plants without any treatment. Epidermis: The outermost layer of the gall, composed of flattened single cells with a thin, semi-transparent appearance, usually bearing hairs (trichomes) on the surface. It is derived from the periderm of normal plant tissue and has stomata distributed on its surface. Outer protective layer: The parenchyma tissue beneath the epidermis, characterized by a light green color. Inner protective layer: Relatively hard sclerenchyma tissue located outside the nutritive layer, with a whitish color. Nutritive layer: The innermost layer lining the larval chamber, composed of thin-walled, densely cytoplasmic cells. It often appears moist and is in direct contact with the larva.

For gall tissue layers, each biological replicate for a given layer was obtained from a distinct set of chestnut galls. No single gall was used to isolate more than one tissue layer. For example, epidermis samples were collected from one group of galls, outer protective layer from a separate group, inner protective layer from another group, and nutritive layer from yet another group. For insect developmental stages (larva, pupa, adult), each sample required pooling multiple individuals to achieve a minimum fresh weight of 0.6 g for cytokinin extraction. Due to the small size of the galls, each biological replicate for each layer was pooled from multiple individual galls to obtain sufficient material for analysis.

### 2.2. Cytokinin Analysis

Reagents and standards. Unlabeled cytokinin standards (cis-zeatin, CZ; dl-dihydrozeatin riboside, DZR; trans-zeatin riboside, TZR; trans-zeatin, TZ; N^6^-(Δ^2^-isopentenyl)adenine, IP; and N^6^-isopentenyladenosine, IPR) and their deuterated internal standards ([^2^H_6_]-CZ, [^2^H_6_]-DZR, [^2^H_6_]-TZR, [^2^H_6_]-TZ, [^2^H_6_]-IP, [^2^H_6_]-IPR) were purchased from Shanghai Zhenzhun Biotechnology Co., Ltd. (Shanghai, China). HPLC-grade methanol, acetonitrile, and formic acid were obtained from Merck (Darmstadt, Germany) and Thermo Fisher Scientific (Waltham, MA, USA). C18 sorbent (40–60 μm) was from ANPEL Laboratory Technologies (Shanghai, China). Ultrapure water was used throughout.

Sample extraction and purification. Approximately 1 g of liquid-nitrogen-ground sample (or pooled tissue equivalent to 0.5 g fresh weight) was weighed into a 50 mL centrifuge tube. The sample was mixed with 10 mL of acetonitrile containing 4 μL of internal standard mother solution (1 μg/mL) and extracted overnight at 4 °C. After centrifugation at 12,000× *g* for 5 min, the supernatant was collected. The pellet was re-extracted twice with 5 mL of acetonitrile, and all supernatants were combined. To remove pigments and non-polar interferents, the combined supernatant was subjected to dispersive solid-phase extraction (d-SPE) with 15–40 mg of C18 sorbent. The mixture was vortexed for 30 s, centrifuged at 10,000× *g* for 5 min, and the clarified supernatant was evaporated to dryness under nitrogen at 40 °C. The residue was reconstituted in 400 μL of methanol, filtered through a 0.22 μm organic membrane, and stored at −20 °C until analysis.

Calibration standards. Internal standard mother solution (1 μg/mL) was prepared by mixing 2 μL of each deuterated internal standard stock solution (500 μg/mL) with 992 μL of methanol. External standard mother solution (1 μg/mL) was prepared by mixing 2 μL of each unlabeled standard stock solution (500 μg/mL) with 986 μL of methanol. Calibration solutions were prepared by adding 0.1, 0.2, 0.5, 2, 5, 20, 50, and 200 μL of the external standard mother solution to tubes containing decreasing volumes of methanol (979.9 to 880 μL), followed by 10 μL of internal standard mother solution per tube. Final concentrations ranged from 0.1 to 200 ng/mL for unlabeled hormones, with a constant internal standard concentration of 10 ng/mL.

UPLC-MS/MS conditions. Chromatographic separation was performed on a Poroshell 120 SB C18 reversed-phase column (2.1 × 150 mm, 2.7 μm) at 30 °C. Mobile phases were A: methanol (0.1% formic acid) and B: water (0.1% formic acid) at a flow rate of 0.3 mL/min. The gradient program is provided in Table 1. Injection volume was 2 μL. Mass spectrometry was conducted with electrospray ionization (ESI) in positive and negative ion modes separately. Curtain gas: 15 psi; ion spray voltage: +4500 V (positive)/−4000 V (negative); nebulizer gas (Gas 1): 65 psi; heater gas (Gas 2): 70 psi; source temperature: 400 °C. Ionization mode: Electrospray ionization (ESI) in positive and negative ion modes separately monitored. Scan type: Multiple reaction monitoring (MRM). Curtain gas: 15 psi. Ion spray voltage: +4500 V (positive mode)/−4000 V (negative mode). Nebulizer gas (Gas 1): 65 psi. Heater gas (Gas 2): 70 psi. Source temperature: 400 °C.

### 2.3. Statistical Analysis

Statistical tests used are reported with the results. The results are presented as the mean ± standard error from 3–6 replicate experiments.

All statistical analyses were performed using R software (version 4.2.1, R Core Team, 2022) with the packages ggpubr, dplyr, and rstatix. A significance level of α = 0.05 was used for all tests. Data normality was assessed using the Shapiro–Wilk test, and homogeneity of variances was checked using Levene’s test. Because most cytokinin datasets deviated from normality and showed heteroscedasticity, non-parametric methods were employed. For comparisons across multiple independent groups (e.g., among the ten sample types: normal leaf, four gall tissue layers, larva, pupa, adult head, adult thorax, and adult abdomen), each of the six cytokinins (TZR, DZR, CZ, TZ, IP, IPR) was analyzed separately. For each cytokinin, the Kruskal–Wallis test was applied to determine whether significant differences existed among the ten groups. When the Kruskal–Wallis test was significant (*p* < 0.05), pairwise multiple comparisons were performed using Dunn’s post hoc test with Bonferroni correction for multiple testing.

## 3. Results

### Differences in Hormone Content Among Different Samples

The Kruskal–Wallis test revealed significant differences in the contents of six cytokinins among the ten samples: (TZR: H = 34.23, df = 9, *p* < 0.001; DZR: H = 32.26, df = 9, *p* < 0.001; CZ: H = 31.79, df = 9, *p* < 0.001; TZ: H = 33.08, df = 9, *p* < 0.001; IP: H = 33.29, df = 9, *p* < 0.001 and IPR: H = 34.58, df = 9, *p* < 0.001).

Regarding the types of cytokinins, among the six cytokinins detected, IPR and TZR were the predominant accumulation forms. In terms of tissue distribution, IPR was mainly concentrated in larvae (63.12 ± 2.55 ng/g), adult abdomen (37.27 ± 0.02 ng/g), and adult thorax (34.81 ± 0.07 ng/g), with levels significantly higher than those in normal tissue and outer protective layer (*p* < 0.05, Dunn’s test with Bonferroni correction; Table 2). However, no significant difference was found between larvae, abdomen, and thorax. TZR levels were highest in the nutritive layer (4.90 ± 1.29 ng/g) and larvae (3.94 ± 0.69 ng/g), but were almost undetectable in the pupal stage (0.01 ± 0.01 ng/g). However, the level of TZ detected in larvae (3.96 ± 0.34 ng/g) is noteworthy given the high biological activity of trans-zeatin. In contrast, TZ showed marked accumulation in larvae (3.96 ± 0.59 ng/g). These patterns suggest that different tissues possess specific cytokinin metabolic capabilities, reflecting functional specialization among tissues.

## 4. Discussion

Our results show that in *Dryocosmus kuriphilus*, the most abundant cytokinin in larvae (63.12 ± 2.55 ng/g IPR) and in adults (22 to 37 ng/g IPR) is isopentenyladenosine (IPR), followed by trans-zeatin (TZ) in larvae (3.96 ± 0.34 ng/g) and TZR in larvae (3.94 ± 0.69 ng/g). These values are comparable to or higher than those reported for other gall-inducing insects. For example, IPR in *Trichilogaster acaciaelongifoliae* larvae was reported as 2 ng/g, and in *Eurosta solidaginis* IP content reached 50 ng/g [19,29]. The high IPR level in our study is consistent with the hypothesis that IP-type cytokinins are predominant in many gall-forming Hymenoptera.

In contrast, normal chestnut leaves contained very low or undetectable levels of most CKs (e.g., TZ 0.07 ± 0.01 ng/g, IPR 0.24 ± 0.01 ng/g). Notably, zeatin-type CKs (TZ and TZR) were detected in galls and wasp tissues but not in normal leaves. This pattern is similar to observations in *Pontania pacifica* and *Tamalia coweni*, where CKs were enriched in insect-associated tissues [25]. However, our data alone cannot distinguish whether these CKs are synthesized *de novo* by the wasp or accumulated from plant tissues during feeding. The fact that normal leaves lack zeatin-type CKs while galls and wasps contain them is consistent with both gall-induced plant synthesis and insect biosynthetic capacity.

Although the presence of high IPR and TZR in larvae and adults might suggest insect biosynthesis, an equally plausible explanation is that the wasp ingests plant material that contains elevated CKs (induced by gall formation) and subsequently accumulates them in its body. Without functional genetic evidence (e.g., silencing of putative insect *IPT* or *LOG* homologs) or isotope tracing experiments, we cannot rule out the plant-to-insect transfer pathway.

A key finding is the high concentration of IPR in the adult head (22.09 ± 0.10 ng/g), thorax (34.81 ± 0.07 ng/g) and abdomen (37.27 ± 0.02 ng/g). While the high IPR and TZR levels in the abdomen are consistent with the hypothesis that CKs are injected during oviposition to initiate gall formation [8], the equally high values in the head and thorax require explanation.

We propose several non-exclusive interpretations: (i) Metabolic and physiological demands—IPR may support high energy turnover in flight muscles (thorax) and neural activity (head). In insects, cytokinins have been implicated in stress responses and energy metabolism [6]. (ii) Tissue specific accumulation without direct gall-inducing function—IPR in the head and thorax could simply reflect constitutive expression of CK related enzymes in these tissues, without being secreted into host plants. Future microdissection of specific organs (e.g., salivary glands, neural tissues, flight muscles) and functional assays are needed to clarify the biological significance of high IPR in the head and thorax.

The drastic decrease in all six CKs from larvae to pupae (e.g., IPR from 63.12 to 0.85 ng/g) supports the idea that CK levels are coupled with feeding activity and metabolic demand. During the non-feeding pupal stage, low CKs may reduce energy expenditure and avoid triggering plant defense responses [4,6]. The reappearance of high IPR in adults, especially in the abdomen, is consistent with a role in oviposition-related gall induction. However, because of the small body size (2.5–3.0 mm), we could not microdissect the ovipositor glands for direct CK quantification. A further limitation is the inability to localize cytokinins to specific tissues or cell types within the insect body, due to the relatively large sample mass required for UPLC–MS/MS analysis. Microdissection combined with immunohistochemistry represents a promising complementary approach for spatial mapping of cytokinins when tissue quantities are limiting [21,30]. Future studies employing these techniques will be valuable to determine, for example, whether CKs are specifically concentrated in the ovipositor glands (supporting the oviposition induction hypothesis).

## Figures and Tables

**Table 1:** Gradient elution program.

Time (min)	A (%)	B (%)
0–1	20	80
1–3	20 → 50	80 → 50
3–9	50 → 80	50 → 20
9–10.5	80	20
10.5–10.6	80 → 20	20 → 80
10.6–13.5	20	80

**Table 2:** Concentrations of cytokinins (CK) including trans-zeatin riboside (TZR), dihydrozeatin (DZR), *cis*-Zeatin (CZ), *trans*-Zeatin (TZ), isopentenyladenosine (IPR), and isopentyladenine (IP) in galls and whole-body fresh weight (0.1 g) of *Dryocosmus kuriphilus*, expressed as mean ± standard error (SE) in ng/g FW. Biological replicates: *n* = 3 for all sample types. Different letters within the same column indicate significant differences (*p* < 0.05, Kruskal–Wallis test followed by Dunn’s post hoc test with Bonferroni correction), while the same letter denotes no significant difference.

	*cis*-Zeatin	*cis*-Zeatin Group	*trans*-Zeatin	*trans*-Zeatin Group	TZR	TZR Group	DZR	DZR Group	IP	IP Group	IPR	IPR Group
Normal tissue	0.01 ± 0.01	b	0.07 ± 0.01	bc	0.11 ± 0.02	b	0.03 ± 0.01	bc	0.39 ± 0.01	ab	0.24 ± 0.01	b
Nutritive layer	0.01 ± 0.01	bc	0.09 ± 0.01	abc	4.90 ± 1.29	a	0.06 ± 0.01	abc	0.28 ± 0.02	ab	3.07 ± 0.04	ab
Inner protective layer	0.05 ± 0.01	abc	0.08 ± 0.01	abc	0.56 ± 0.03	ab	0.09 ± 0.01	abc	0.17 ± 0.01	ab	0.44 ± 0.08	ab
Outer protective layer	0.02 ± 0.01	abc	0.05 ± 0.01	bc	0.23 ± 0.08	ab	0.06 ± 0.01	abc	0.17 ± 0.01	ab	0.15 ± 0.03	b
Epidermis	0.07 ± 0.01	a	0.10 ± 0.01	abc	0.42 ± 0.01	ab	0.10 ± 0.01	a	0.18 ± 0.01	ab	3.92 ± 0.15	ab
Larvae	0.02 ± 0.01	abc	3.96 ± 0.34	a	3.94 ± 0.69	a	0.05 ± 0.01	abc	0.17 ± 0.01	ab	63.12 ± 2.55	a
Pupa	0.03 ± 0.01	abc	0.01 ± 0.01	b	0.01 ± 0.01	b	0.09 ± 0.01	ab	0.16 ± 0.01	a	0.85 ± 0.01	ab
Adult head	0.07 ± 0.01	ac	0.14 ± 0.01	abc	0.84 ± 0.01	ab	0.02 ± 0.01	c	2.82 ± 0.01	b	22.09 ± 0.10	ab
Adult thorax	0.05 ± 0.01	abc	0.20 ± 0.01	abc	1.94 ± 0.01	ab	0.06 ± 0.01	abc	1.85 ± 0.01	b	34.81 ± 0.07	ab
Adult abdomen	0.06 ± 0.01	abc	0.21 ± 0.01	ac	2.07 ± 0.01	ab	0.07 ± 0.01	abc	1.82 ± 0.01	ab	37.27 ± 0.02	a

## Data Availability

The original contributions presented in this study are included in the article. Further inquiries can be directed to the corresponding author.
